# Pilot Study: Nutritional and Preclinical Safety Investigation of Fermented Hispidin-Enriched *Sanghuangporus sanghuang* Mycelia: A Promising Functional Food Material to Improve Sleep

**DOI:** 10.3389/fnut.2021.788965

**Published:** 2022-01-17

**Authors:** I-Chen Li, Fang-Chia Chang, Ching-Chuan Kuo, Hsin-Tung Chu, Tsung-Ju Li, Chin-Chu Chen

**Affiliations:** ^1^Biotech Research Institute, Grape King Bio Ltd., Taoyuan, Taiwan; ^2^Department of Veterinary Medicine, School of Veterinary Medicine, National Taiwan University, Taipei, Taiwan; ^3^Graduate Institute of Brain and Mind Sciences, College of Medicine, National Taiwan University, Taipei, Taiwan; ^4^Graduate Institute of Acupuncture Science, College of Chinese Medicine, China Medical University, Taichung, Taiwan; ^5^Institute of Biotechnology and Pharmaceutical Research, National Health Research Institutes, Miaoli, Taiwan; ^6^Institute of Food Science and Technology, National Taiwan University, Taipei, Taiwan; ^7^Department of Bioscience Technology, Chung Yuan Christian University, Taoyuan, Taiwan; ^8^Department of Food Science, Nutrition and Nutraceutical Biotechnology, Shih Chien University, Taipei, Taiwan

**Keywords:** *Sanghuangporus sanghuang* mycelia, hispidin, NOAEL, sleep, Nrf2

## Abstract

Sleep disturbances have been the hallmark of the recent coronavirus disease 2019 pandemic. Studies have shown that once sleep is disrupted, it can lead to psychological and physical health issues which can, in turn, disrupt circadian rhythm and induce further sleep disruption. As consumers are trying to establish healthy routines, nutritional and preclinical safety investigation of fermented hispidin-enriched *Sanghuangporus sanghuang* mycelia (GKSS) as a novel food material for spontaneous sleep in Sprague-Dawley rats is conducted for the first time. Results showed that the nutritional analysis of GKSS including moisture, ash, crude lipid, crude protein, carbohydrate, and energy were found to be 2.4 ± 0.3%, 8.0 ± 2.5%, 1.7 ± 0.3%, 22.9 ± 1.2%, 65.1 ± 3.1%, and 367.1 ± 10.2 kcal/100 g respectively. In the 28-day repeated-dose oral toxicity study, only Sprague-Dawley male rats receiving 5 g/kg showed a slight decrease in feed consumption at week 3, but no associated clinical signs of toxicity or significant weight loss were observed. Although a significant reduction of the platelet count was found in mid- and high-dose GKSS treated male groups, such changes were noted to be within the normal range and were not correlated with relative spleen weight changes. Hence, the no observed adverse effect level (NOAEL) of GKSS was identified to be higher than 5 g/kg in rats. After the safety of GKSS is confirmed, the sleep-promoting effect of GKSS ethanolic extract enriched with hispidin was further assessed. Despite 75 mg/kg of GKSS ethanolic extract does not affect wakefulness, rapid eye movement (REM) sleep and non-REM (NREM) sleep, GKSS ethanolic extract at 150 mg/kg significantly decreased wakefulness and enhanced NREM and REM sleep. Interestingly, such effects seem to be mediated through anti-inflammatory activities via NF-E2-related factor-2 (Nrf2) signaling pathway. Taken together, these findings provide the preliminary evidence to studies support the claims suggesting that GKSS contained useful phytochemical hispidin could be considered as and is safe to use as a functional food agent or nutraceutical for relieving sleep problems mediated by Nrf2 pathway, which the results are useful for future clinical pilot study.

## Introduction

From December 2019, a modified coronavirus called severe acute respiratory syndrome coronavirus 2 (SARS-CoV-2) has entered the human population and spread rapidly to almost every region of the globe, leading to the coronavirus disease 2019 (COVID-19) pandemic. After the outbreak, numerous studies of COVID-19-associated sleep disorders have been reported (1). A study conducted in China using a self-reported psychological and sleep online survey showed that both healthy and mental health disorders adults have a significantly increased prevalence of anxiety, depression, and insomnia when compared to the pre-COVID-19 period (all *p-*value < 0.001) (2). In another survey including 2,291 Italian respondents, 57.1% reported poor sleep quality, 32.1% high anxiety, 41.8% high distress, and 7.6% reported post-traumatic stress disorder symptomatology linked to COVID-19 (3). Previous studies have shown that anxiety and stress are closely linked and are frequently connected to endless cycles of stress and insomnia (4). Moreover, insufficient sleep has been linked to 7 of the 15 leading causes of death in the United States, including accidents, cardiovascular disease, cerebrovascular disease, diabetes, hypertension, malignant neoplasm, and septicemia (5). As sleep disorders became a hallmark of the COVID-19 pandemic, it is now a public health concern that needs to be addressed.

Epidemiological studies suggested that sleep deprivation activates nuclear factor (NF)-κB, and induces increases in circulating levels of inflammatory markers, such as C-reactive protein (CRP), interleukin 6 (IL-6), and tumor necrosis factor-α (TNF-α) (6). Conversely, when low doses of IL-6 were injected in humans, slow-wave sleep was found to decrease during the first half of sleep and rapid eye movement sleep was found to significantly decrease during the entire nocturnal sleep time (7). As the relationship between sleep and inflammation is likely bidirectional, modulation of inflammation might reverse sleep disturbance, improve sleep, and promote sleep health. Emerging evidence supports this possibility. In a cohort of Italian adults, individuals who intake diets with a high dietary inflammatory index (DII) were less likely to have adequate sleep quality (8). In another study, participants with anti-inflammatory DII diets for 3 months were found to decrease wake-after-sleep-onset and improve sleep efficiency when compared to participants with pro-inflammatory DII diets. Hence, an intervention that aims to lower chronic systemic inflammation may be a promising strategy to promote sleep duration and quality.

*Phellinus linteus*, commonly known as “sanghuang” in China, “meshimakobu” in Japan, and “sangwhang” in Korea, is a popular medicinally valuable mushroom that has been used as an ingredient to alleviate sickness in humans for more than thousands of years (9). In modern pharmacological studies, *P. linteus* was found to possesses antitumor, anti-inflammatory, antiviral, antimicrobial, antioxidant, antidiabetic, hepatoprotective, neuroprotective and immunomodulatory activities (10). Among the phytochemical constituents of *P. linteus*, the yellow polyphenol pigment hispidin was best studied. Research has indicated that hispidin displays antioxidant (11), anti-obesity (12), anti-cancer (13), anti-viral (14), anti-allergic (15), and anti-gout effects (16). Moreover, in a recent study, hispidin was found to exhibit anti-inflammatory activity by suppressing reactive oxygen species-mediated NF-κB pathway in mouse macrophage cells (17). Activation of inflammatory signaling pathways like the inflammasome or NF-κB can lead to a release of acute-phase cytokines such as IL-1, IL-6, and TNF (18). In rats, 25 ng of IL-1 was found to suppress electroencephalogram (EEG) slow-wave amplitudes at night and decrease rapid-eye-movement (REM) sleep during the day. In healthy participants, studies have shown that subcutaneous administration of cytokine IL-6 before sleep initiation resulted in a delayed REM sleep latency and reduced REM sleep amount (7). Considering that both *P. linteus* and hispidin possess anti-inflammatory activities, *P. linteus* enriched with hispidin may play a role in sleep benefits. However, only *P. linteus* from extreme environments can synthesis small organic molecules such as hispidin with specific biological activities (19, 20). Moreover, the association of sleep with *P*. *linteus* enriched with hispidin has not yet been explored. Hence, one of the objectives of the present study is to evaluate the sedative and hypnotic effects of *P. linteus* enriched with hispidin.

Moreover, despite *P. linteus* holds promising pharmacological potential, its identification has been called into question due to the inadequate morphological analyses within allied species (21). In Asia, *P. linteus* has been adopted as the scientific binomial for the “sanghuang” species for centuries. However, with the advent of DNA sequencing, studies have found that *P. linteus* is only distributed in Central America and not in Asia (22). Moreover, the genuine “sanghuang” was a new and previously undescribed species, named *Inonotus sanghuang* in 2012, which grows solely on *Morus* tree (22). By 2016, a new genus *Sanghuangporus* was proposed to accommodate *I. sanghuang* and other closely related species, and *Sanghuangporus sanghuang* thus became the scientific binomial for the “sanghuang” species (23). As inaccurate taxonomy may result in conflicting reports on if “sanghuang” species are edible or not, there is a need to build a scientific consensus on the safety of true “sanghuang” strains for human consumption. In our previous study, the “sanghuang” strain was authenticated and identified as *Sanghuangporus sanghuang* based on detailed molecular phylogenetic studies (20). In addition, neither mutagenicity nor abnormal changes were caused by 3 mg/g hispidin-enriched *S. sanghuang* mycelia treatment *in vitro* and *in vivo*, respectively. To our knowledge, a long-term oral toxicity study of *S. sanghuang* has not been evaluated. Therefore, as our follow-up study, the second objective of the present study is to determine the sub-chronic toxicity profile of 3 mg/g hispidin-enriched *S. sanghuang* after 28 days of consecutive exposure in Sprague-Dawley rats.

## Materials and Methods

### Sample Preparation, Proximate Composition, Extraction and Hispidin Isolation

Hispidin-enriched *S. sanghuang* mycelia (GKSS) were grown under optimal conditions according to the previous study (20) to enhance the production of hispidin, which was quantified by high-performance liquid chromatography (HPLC) to be 3 mg/g. Following fermentation, the mycelial biomass is separated from the fluid media, dried, and ground to a powder. Proximate analysis of GKSS including crude protein, crude fat, crude fiber, ash, moisture, and carbohydrate were then examined according to the Association of Official Analytical Chemists methods (24). The energy value was calculated by multiplying proteins by 4, carbohydrates by 4, and fats by 9.

According to a previous study, ethanol was the best solvent for obtaining extracts rich in hispidin (25) and hence was selected as the extraction solvent for subsequent experiments. In brief, 50 g of GKSS powder was added to 1000 ml of absolute alcohol, extracted for 1 h in an ultrasonic bath, filtered through Whatman filter paper No. 4, and concentrated through a rotary evaporator (R-220; Büchi Labortechnik AG, Flawil, Switzerland). Dried ethanolic extract was then further reconstituted in ethanol to obtain a final concentration of 100 mg/mL and filtered through a sterilized 0.22 μm syringe before exposure to the animals.

Hispidin was isolated according to a previous study with slight modification (26). Solvents such as methanol, formic acid, acetonitrile, and n-hexane used for extraction and chromatographic separation were analytical grade (Merck, Darmstadt, Germany). In brief, the ethanolic extract was further partitioned with Hexane −90% MeOH (2:1) to afford a hexane and a 90% MeOH layer. The hexane layer was re-extract with hexane, vortex and centrifuge at 3,600 × g for 5 min. After the upper layer was discarded, the extraction steps were repeated two additional times and concentrated through a rotary evaporator to afford hispidin. Ultra-Performance Liquid Chromatography (UPLC) analysis of hispidin was executed with a Kinetex Core-Shell C_18_ analytical column (3 × 100 mm, particle size 1.7 μm; Phenomenex, CA, USA) on an Agilent 1,290 system (Agilent Technologies, Santa Clara, CA, USA). Separation was performed at 40 °C with a mobile phase gradient of 0.1 % formic acid (A) and acetonitrile (B). The gradient elution had the following profile: 0–10 min, 15–40 % (B); 10–10.1 min, 40–100 % (B); 10.1–12 min, 100–100 % (B); 12–12.5 min, 100–15% (B); 12.5–15 min, 15–15 % (B). The retention time of hispidin was 3.36 min at a flow rate of 1.0 mL/min with a scanning UV wavelength at 380 nm.

### Experimental Animals

Sprague Dawley rats procured from BioLASCO Taiwan Co., Ltd. (Taipei, Taiwan) were acclimated and quarantined for 1 week prior to the initiation of the study. The animals were housed in pairs in polypropylene cages in a well-ventilated room (10–15 air changes/h) under an ambient temperature of 22 ± 3°C and 55 ± 15% relative humidity, with a 12:12 h light regime (lights on at 6:00 AM, lights off at 6:00 PM). Standard rodent diet (MGF; Oriental Yeast Co., Ltd., Tokyo, Japan) and purified water were available *ad libitum*.

### 28-Day Oral Toxicity Study

This animal study was implemented in accordance with guidelines of the Organization for Economic Cooperation and Development Guideline 407 “Repeated Dose 28-day Oral Toxicity Study in Rodents” (27) and approved by the Institutional Animal Care and Use Committee (IACUC No. 102-9n). As our earlier study on acute oral toxicity indicated that acute oral LD_50_ of GKSS was greater than 12 g/kg in Sprague-Dawley rats (20), three treatment doses of 1 g/kg (low dose), 2 g/kg (mid-dose) and 5 g/kg (high-dose) were thus selected for this study. After 80 healthy 5 weeks-old rats of both genders with an average body weight ranging from 180 to 265 g had their health status evaluated, they were randomly assigned into one vehicle control group and three treatment groups. GKSS was orally administered by gavage. Throughout the experiment, signs of toxicity, mortality and morbidity were monitored daily. Detailed clinical signs including changes in skin, fur, eyes, mucous membranes, occurrence of secretions and excretions, and autonomic activities were recorded. The body weight and feed intake were recorded weekly while ophthalmological examination was performed prior to initiation of dosing and before sacrifice. Before the scheduled sacrifice, urine samples were collected from surviving rats for 16 h and the following parameters were evaluated with a urine analyzer (PU-4010, ARKRAY, Japan): specific gravity (SG), color, protein, urobilinogen, pH, ketone, bilirubin, glucose, nitrite, and occult blood. Urine samples were then centrifuged to obtain sediments, which were examined microscopically for the presence of white blood cell (WBC), red blood cell (RBC), epithelial cell (EP), crystals, and microbes.

At the end of the stipulated experimental period, rats were fasted overnight and anesthetized by inhalation of carbon dioxide. Blood was drawn from the abdominal aorta into EDTA-containing and non-anticoagulant-containing collection tubes for hematological and biochemistry analyses, respectively. Hematology was measured using an automatic blood analyzer (Sysmex CA-1500, Kobe, Japan) with the following parameters including RBC, WBC, hematocrit, hemoglobin, platelet count, mean corpuscular volume (MCV), mean corpuscular hemoglobin (MCH), mean corpuscular hemoglobin concentration (MCHC), lymphocyte, neutrophil, monocyte, eosinophil, basophil and prothrombin time (PT). On the other hand, serum biochemistry parameters such as alkaline phosphatase (ALP), aspartate aminotransferase (AST), alanine aminotransferase (ALT), albumin, total protein, total bilirubin, creatinine, blood urea nitrogen (BUN), glucose, cholesterol, triglyceride, phosphorus, calcium, chloride, potassium and sodium, were examined using an automated biochemistry analyzer (7070, Hitachi Ltd., Tokyo, Japan).

After sacrifice, a complete gross necropsy including examination of external surfaces, orifices, cranial cavities, carcass, and organs was performed. Selected organs such as brain, heart, kidneys, livers, spleen, adrenal gland and ovaries/testes were isolated, weighed, fixed and preserved in 10% neutral buffered formalin for histology. 2 μm paraffin wax tissue sections from fixed organs were then stained with hematoxylin-eosin and examined with a microscope (BX51, Olympus, Tokyo, Japan) to detect any lesions.

### Sedative Activity Study

The protocol for sedative activity was approved by the Institutional Animal Care and Use Committee (IACUC approval: NTU107-EL-00182) of National Taiwan University. Following acclimatization, eight-week-old male Sprague Dawley rats weighing 250–300 g were anesthetized with Zoletil (50 mg/kg; Virbac, Carros, France) and xylazine (14.8 mg/kg; Sigma-Aldrich, MO, USA) before being surgically implanted with electroencephalography (EEG) and electromyography (EMG) electrodes as well as a microinjection cannula for polysomnographic recordings. Precisely, two and one EEG screw electrodes were implanted in the frontal and parietal lobes of the right hemisphere and the occipital lobe of the left hemisphere, respectively while two EMG electrodes were implanted in the neck muscle. Once the signals from the EEG and EMG electrodes had been detected, they were fed into an amplifier (Colbourn Instruments, Lehigh Valley, PA, USA; model V75-01), filtered from 0.1 and 40 Hz and digitized (NI PCI-6033E; National Instruments, Austin, TX, USA) at 128 Hz. Following 7 days of postsurgical recovery, rats were randomly assigned one control and two treatment groups, each comprising six rats. Control group (5.5% ethanol) and two doses (75 and 150 mg/kg; suspended in 5.5% ethanol) of GKSS ethanolic extract were administered orally 20 min before the onset of the dark period. To easily observe the hypnotic effect, the substance was administered prior to the dark period as rats are generally active during the dark period and somnolent during the light period. The sleep-wake activity patterns were then recorded consecutively for 24 h in 12-s epochs to determine different sleep stages (wakefulness, non-REM (NREM) sleep, REM sleep) based on previously defined criteria (28).

### NF-E2-Related Factor-2 (Nrf2) Luciferase Reporter Assay

Cells were transfected with Nrf2 pathway responsive gene, antioxidant-responsive element (ARE) pGL3 vector and internal control pRL-TK (*Renilla* luciferase) plasmids according to our previous study (29). In brief, stable ARE-driven luciferase reporter cells HSC3-ARE9 were cultured in MEM (GibcoBRL, NY, USA), supplemented with 10% fetal bovine serum, 1 mM sodium pyruvate, penicillin (100 U/mL), streptomycin (100 μg/mL), and 2 mM L-glutamine, at 37°C in a humidified incubator containing 5% CO_2_. For cell viability assay, 2.5 × 10^4^ cells in 100 μL of culturing medium were seeded onto 96-well plates for 24 h before treatments. Cells were then treated with appropriated concentrations (12.5, 25, 50, and 100 μg/mL) of hispidin and GKSS ethanolic extract (37.5, 75, 150, and 300 μg/mL) for 24 h. 20 μL of MTT (5 mg/mL) was added into each sample and incubated for 4 h, under 5% CO_2_ and 37°C. 200 μL of DMSO was subsequently added into each sample and gently shaked for 10 min at room temperature. Absorbance was measured at 560 nm using a Multiskan™ GO Microplate Spectrophotometer (Thermo Scientific, MA, USA). For luciferase activity assay, HSC3-ARE9 cells at a density of 2.5 × 10^4^ per well were seeded in 96-well plates and incubated at 37°C for 16 h. Cells were treated with various concentrations of hispidin, GKSS ethanolic extract and tert-butylhydroquinone (t-BHQ, #112941, Sigma, MO, USA; positive control) for 24 h, and then cell lysates were prepared for assessment of luciferase activity. Luciferase activities were quantified using Steady-Glo® Luciferase Assay System (Promega, WI, USA), according to the manufacturer's protocol.

### Anti-inflammatory Activities

RAW 264.7 macrophages (ATCC TIB-71™, VA, USA) were grown at a density of 2.5 × 10^5^ cells/mL in a 96-well plate, pretreated with various doses of isolated hispidin (50, 75, and 100 μg/mL) and GKSS (75, 150 and 300 μg/mL) for 1 h before stimulated with 100 ng/mL of LPS for 24 h. After incubation, the cells were centrifuged and the supernatant was harvested. Cell viability was examined using MTT assay. The interleukin-6 (#88-7064-88, Thermo Fisher Scientific, MA, USA) and tumor necrosis factor-alpha (#88-7324, Thermo Fisher Scientific, MA, USA) concentrations in cell culture supernatants were then measured via ELISA kit according to the manufacture's protocol. As nitric oxide (NO) is unstable, the quantitative of NO was indirectly determined through the measurement of nitrite (NO_2_) levels. Briefly, 100 μL of supernatant was mixed with an equal volume of Griess reagent for 10 min and the absorbance at 550 nm was measured using a microplate spectrophotometer (Bio-RAD Laboratories, CA, USA). The amount of NO concentration was then calculated by referring to a sodium nitrite standard curve.

### Statistical Analysis

In the 28-day oral toxicity study and Nrf2 luciferase reporter assay, data are given as mean ± standard deviation (SD). For 28-day oral toxicity study, data were analyzed using a one-way analysis of variance (ANOVA) method, followed by Duncan's multiple range test (SPSS, version 12.0, Illinois, Chicago, USA). For Nrf2 luciferase reporter assay and anti-inflammatory activities, one-way ANOVA was performed, followed by pairwise Tukey test. For the sedative activity study, data are given as mean ± standard error of the mean (SEM), respectively and were analyzed using ANOVA. In all cases, *p-*value below 0.05 is considered statistically significant.

## Results

### Nutritional Investigation of GKSS

The proximate composition of 100 g GKSS powder is shown in Table 1. Parameters studied in the proximate analysis include moisture, ash, crude lipid, crude protein, carbohydrate, and energy, which were found to be 2.4 ± 0.3%, 8.0 ± 2.5%, 1.7 ± 0.3%, 22.9 ± 1.2%, 65.1 ± 3.1%, and 367.1 ± 10.2 kcal/100 g respectively.

**Table 1:** Proximate analysis of GKSS powder.

		**Proximate analysis (%)**	
Moisture	2.4	±	0.3
Ash	1.9	±	0.0
Fiber	6.1	±	2.5
Crude lipid	1.7	±	0.3
Crude protein	22.9	±	1.2
Carbohydrate	65.1	±	3.1
Energy (kcal/100g)	367.1	±	10.2

*Each value is expressed in mean ± SD (n = 2)*.

### Preclinical Safety Investigation of GKSS

**Figure 1:**
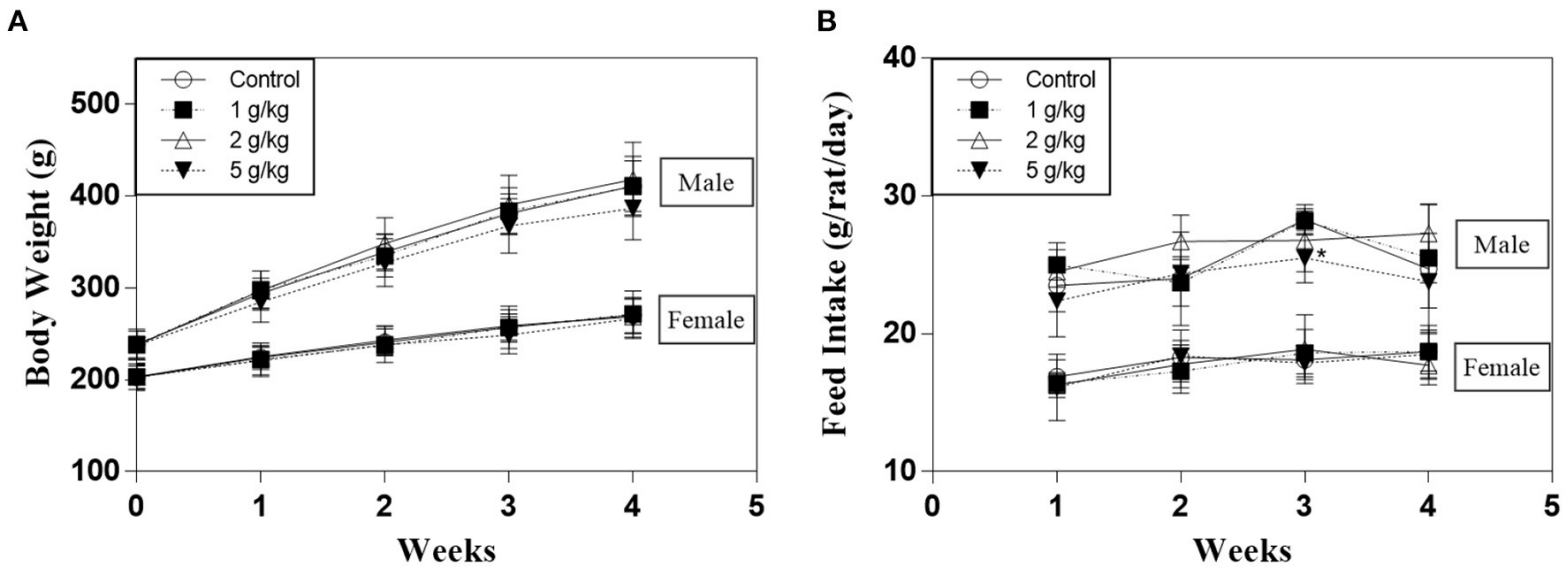
**(A)** Body weight and **(B)** feed intake of male and female Sprague-Dawley rats during the course of study. Data were expressed as mean ± SD (*n* = 10). *p < 0.05 indicates significant differences compared with the control group.

After ingesting GKSS for 28 days, all the animals were in good condition and showed no symptoms of toxicity, morbidity or mortality in both sexes of rats (Supplementary Table 1). There were no abnormal urinalysis findings in any of the animals (data not shown). Except for male rats in the high dose group, which consumed significantly less (25.5 ± 1.8) than the control group (28.3 ± 1.1) during the third week of the study (*p* < 0.05), GKSS did not cause significant changes in the body weight and feed intake during the observation period as depicted in Figure 1.

Blood hematology values in male and female rats are shown in Table 2. There were statistically significant decreases (*p* < 0.05) in the absolute value of platelet counts in the mid- and high-dose test male groups when compared to the control group (Table 2). However, no statistically significant alteration was found in other parameters investigated.

**Table 2:** Hematological parameters of male and female Sprague-Dawley rats after 28 days daily oral administration of GKSS.

**Parameters**	**Control (Distilled water)**	**GKSS**		
		**1 g/kg**	**2 g/kg**	**5 g/kg**
**Male**				
WBC (10^3^/μl)	14.7 ± 3.7	14.4 ± 3.4	14.2 ± 3.8	14.7 ± 2.9
RBC (10^6^/μl)	8.89 ± 0.47	8.83 ± 0.39	8.83 ± 0.72	8.86 ± 0.43
Hemoglobin (g/dL)	16.6 ± 0.7	16.6 ± 0.6	16.8 ± 1.0	16.8 ± 0.5
Hematocrit (%)	48.6 ± 2.3	48.9 ± 1.8	49.3 ± 2.7	49.4 ± 1.7
MCV (fL)	54.7 ± 2.1	55.4 ± 2.4	56.0 ± 2.4	55.8 ± 2.0
MCH (pg)	18.7 ± 0.7	18.9 ± 0.6	19.0 ± 0.6	18.9 ± 0.6
MCHC (g/dL)	34.3 ± 0.4	34.0 ± 0.4	34.0 ± 0.5	33.9 ± 0.3
Platelet (10^3^/μl)	1,146.8 ± 223.9	1,039.3 ± 128.7	916.5 ± 152.1^*^	973.1 ± 176.6^*^
Neutrophil (%)	17.0 ± 6.8	15.5 ± 5.4	16.2 ± 7.6	13.6 ± 5.1
Lymphocyte (%)	77.4 ± 8.2	79.2 ± 6.2	78.4 ± 8.1	81.4 ± 5.3
Monocyte (%)	4.7 ± 1.7	4.2 ± 1.0	4.5 ± 0.9	3.9 ± 0.8
Eosinophil (%)	0.8 ± 0.2	0.9 ± 0.4	0.8 ± 0.2	0.9 ± 0.3
Basophil (%)	0.2 ± 0.1	0.1 ± 0.1	0.2 ± 0.1	0.2 ± 0.1
PT (sec)	14.6 ± 2.4	15.3 ± 1.9	15.3 ± 2.4	14.3 ± 2.2
**Female**				
WBC (10^3^/μl)	10.3 ± 3.1	10.8 ± 2.5	12.1 ± 4.8	10.1 ± 1.4
RBC (10^6^/μl)	8.70 ± 0.32	8.68 ± 0.51	8.80 ± 0.47	8.61 ± 0.32
Hemoglobin (g/dL)	15.9 ± 0.4	15.9 ± 0.4	16.2 ± 0.6	15.8 ± 0.4
Hematocrit (%)	47.0 ± 1.1	46.9 ± 1.2	47.6 ± 1.5	46.6 ± 1.1
MCV (fL)	54.0 ± 1.4	54.2 ± 2.6	54.2 ± 2.2	54.2 ± 1.6
MCH (pg)	18.3 ± 0.4	18.3 ± 0.7	18.4 ± 0.6	18.4 ± 0.4
MCHC (g/dL)	33.9 ± 0.4	33.9 ± 0.5	34.0 ± 0.4	34.0 ± 0.4
Platelet (10^3^/μl)	1,164.0 ± 203.2	1,127.6 ± 199.1	1,055.0 ± 198.0	1,083.7 ± 169.3
Neutrophil (%)	16.5 ± 5.0	14.3 ± 4.1	17.6 ± 6.2	17.8 ± 4.9
Lymphocyte (%)	77.3 ± 5.4	78.4 ± 5.5	76.9 ± 6.6	75.7 ± 5.5
Monocyte (%)	4.5 ± 1.0	5.6 ± 1.9	4.1 ± 1.2	4.9 ± 1.5
Eosinophil (%)	1.5 ± 0.4	1.6 ± 0.8	1.3 ± 0.4	1.4 ± 0.6
Basophil (%)	0.2 ± 0.1	0.2 ± 0.1	0.2 ± 0.1	0.2 ± 0.1
PT (sec)	10.1 ± 0.1	10.1 ± 0.2	10.1 ± 0.2	10.0 ± 0.2

*Data were expressed as mean ± SD (n = 10)*.

* *p < 0.05 indicates significant differences compared with the control group. WBC, white blood cell; RBC, red blood cell; MCV, mean corpuscular volume; MCH, mean corpuscular hemoglobin; MCHC, Mean corpuscular hemoglobin concentration; PT, prothrombin time*.

Table 3 shows the mean biochemical data in male and female rats across the group. In low-dose treated males, GKSS significantly decreased the serum sodium and chloride values (*p* < 0.05) when compared to the control group. In addition, there was a significant decrease (*p* < 0.05) in serum cholesterol for female groups treated with 2 g/kg of GKSS. No significant alterations were observed, however, in other male or female rat biochemical parameters.

**Table 3:** Biochemical parameters of male and female Sprague-Dawley rats after 28 days daily oral administration of GKSS.

**Parameters**	**Control (Distilled water)**	**GKSS**		
		**1 g/kg**	**2 g/kg**	**5 g/kg**
**Male**				
Glucose (mg/dL)	169.7 ± 36.5	197.2 ± 20.9	186.1 ± 18.7	195.8 ± 28.5
BUN (mg/dL)	12.2 ± 0.8	11.5 ± 1.8	12.9 ± 2.2	12.5 ± 1.6
Creatinine (mg/dL)	0.64 ± 0.05	0.63 ± 0.05	0.64 ± 0.05	0.62 ± 0.04
AST (U/L)	94.7 ± 26.0	110.2 ± 19.4	117.5 ± 38.4	98.4 ± 20.3
ALT (U/L)	30.7 ± 6.9	37.0 ± 5.7	34.9 ± 8.8	31.1 ± 7.1
Total protein (g/dL)	5.8 ± 0.5	5.9 ± 0.3	6.0 ± 0.6	5.9 ± 0.5
Albumin (g/dL)	4.0 ± 0.4	4.2 ± 0.1	4.2 ± 0.3	4.0 ± 0.3
ALP (U/L)	144.2 ± 37.3	143.1 ± 31.3	169.3 ± 24.1	144.6 ± 15
Cholesterol (mg/dL)	61.4 ± 15.8	62.0 ± 21.5	70.9 ± 16.5	64.3 ± 21.6
Triglyceride (mg/dL)	54.5 ± 21.9	58.2 ± 29.7	50.5 ± 20.2	51.4 ± 35.8
Calcium (mg/dL)	10.6 ± 0.7	11.2 ± 0.6	11.0 ± 0.6	10.9 ± 0.8
Phosphorus (mg/dL)	10.0 ± 0.5	10.5 ± 1.0	9.9 ± 0.8	9.6 ± 0.7
Sodium (meq/L)	151.2 ± 1.6	144.5 ± 6.7^*^	148.0 ± 5.1	150.7 ± 1.3
Potassium (meq/L)	6.8 ± 0.3	6.7 ± 1.3	6.5 ± 0.7	6.3 ± 0.7
Chloride (meq/L)	97.8 ± 2.0	94.2 ± 3.2^*^	95.2 ± 3.6	96.4 ± 2.3
Globulin (g/dL)	1.9 ± 0.3	1.7 ± 0.2	1.8 ± 0.4	1.9 ± 0.3
Total bilirubin (μg/dL)	0.005 ± 0.002	0.004 ± 0.002	0.005 ± 0.002	0.005 ± 0.002
**Female**				
Glucose (mg/dL)	115.3 ± 33.7	129.3 ± 23.0	131.6 ± 36.5	155.1 ± 42.0
BUN (mg/dL)	12.7 ± 1.5	12.9 ± 1.9	11.1 ± 1.5	11.3 ± 1.9
Creatinine (mg/dL)	0.67 ± 0.07	0.67 ± 0.07	0.62 ± 0.06	0.65 ± 0.07
AST (U/L)	93.8 ± 19.6	113.9 ± 44.1	96.0 ± 22.3	90.0 ± 13.5
ALT (U/L)	24.8 ± 4.9	38.4 ± 34.1	24.2 ± 5.6	24.9 ± 5.1
Total protein (g/dL)	6.3 ± 0.6	6.3 ± 0.7	5.7 ± 0.5	6.2 ± 0.6
Albumin (g/dL)	4.6 ± 0.5	4.5 ± 0.5	4.1 ± 0.4	4.5 ± 0.4
ALP (U/L)	72.0 ± 10.1	66.2 ± 15.6	75.1 ± 11.0	66.3 ± 13.3
Cholesterol (mg/dL)	89.5 ± 20.0	90.3 ± 20.3	67.5 ± 11.4^*^	87.6 ± 15.7
Triglyceride (mg/dL)	53.0 ± 10.2	52.4 ± 11.6	45.2 ± 7.0	56.4 ± 16.1
Calcium (mg/dL)	10.5 ± 0.9	10.6 ± 1.0	9.9 ± 1.0	10.6 ± 0.8
Phosphorus (mg/dL)	9.3 ± 0.5	9.3 ± 1.2	9.0 ± 0.8	9.5 ± 0.8
Sodium (meq/L)	147.3 ± 1.2	147.2 ± 1.1	147.1 ± 2.1	147.4 ± 1.3
Potassium (meq/L)	7.7 ± 1.1	7.5 ± 1.2	7.3 ± 0.7	7.5 ± 0.5
Chloride (meq/L)	98.2 ± 1.1	97.3 ± 1.3	97.7 ± 2.7	98.0 ± 2.0
Globulin (g/dL)	1.8 ± 0.2	1.8 ± 0.3	1.6 ± 0.3	1.7 ± 0.2
Total bilirubin (μg/dL)	0.007 ± 0.003	0.006 ± 0.002	0.008 ± 0.004	0.007 ± 0.002

*Data were expressed as mean ± SD (n = 10)*.

* *p < 0.05 indicates significant differences compared with the control group. AST, aspartate aminotransferase; ALT, alanine aminotransferase; ALP, alkaline phosphatase; BUN, blood urea nitrogen*.

As shown in Table 4, no significant differences in the absolute organ and relative organ weights were noted between the treatment and control groups in both male and female rats throughout the study period. Furthermore, no abnormal gross findings were identified in any of the animals at necropsy (data not shown).

**Table 4:** Absolute (g) and relative weight (g of 100 g body weight) of male and female Sprague-Dawley rats after 28 days daily oral administration of GKSS.

**Organs**	**Control (Distilled water)**	**GKSS**		
		**1 g/kg**	**2 g/kg**	**5 g/kg**
**Male**				
	Absolute weight, g			
Testes	3.35 ± 0.23	3.33 ± 0.22	3.40 ± 0.30	3.39 ± 0.29
Adrenal	0.067 ± 0.007	0.061 ± 0.011	0.065 ± 0.009	0.070 ± 0.007
Spleen	0.669 ± 0.124	0.716 ± 0.107	0.744 ± 0.165	0.629 ± 0.073
Kidney	3.17 ± 0.41	3.34 ± 0.31	3.22 ± 0.34	3.06 ± 0.28
Heart	1.55 ± 0.19	1.59 ± 0.06	1.55 ± 0.16	1.49 ± 0.11
Brain	2.05 ± 0.07	2.00 ± 0.04	2.00 ± 0.06	2.00 ± 0.07
Liver	12.4 ± 1.8	12.4 ± 1.4	12.8 ± 1.7	11.8 ± 1.4
	Relative weight, g/100 g body weight			
Testes	0.889 ± 0.064	0.880 ± 0.078	0.884 ± 0.088	0.959 ± 0.111
Adrenal	0.018 ± 0.002	0.016 ± 0.003	0.017 ± 0.002	0.020 ± 0.004
Spleen	0.177 ± 0.026	0.188 ± 0.022	0.191 ± 0.028	0.177 ± 0.019
Kidney	0.839 ± 0.059	0.879 ± 0.050	0.833 ± 0.042	0.862 ± 0.080
Heart	0.409 ± 0.037	0.419 ± 0.027	0.400 ± 0.019	0.419 ± 0.034
Brain	0.546 ± 0.033	0.527 ± 0.029	0.522 ± 0.049	0.565 ± 0.059
Liver	3.27 ± 0.29	3.26 ± 0.20	3.31 ± 0.20	3.28 ± 0.15
**Female**				
	Absolute weight, g			
Ovary	0.093 ± 0.013	0.085 ± 0.012	0.101 ± 0.018	0.092 ± 0.021
Adrenal	0.070 ± 0.012	0.068 ± 0.007	0.073 ± 0.009	0.069 ± 0.005
Spleen	0.554 ± 0.060	0.540 ± 0.069	0.552 ± 0.087	0.515 ± 0.082
Kidney	2.10 ± 0.19	2.07 ± 0.20	2.19 ± 0.15	2.10 ± 0.18
Heart	1.04 ± 0.12	1.01 ± 0.13	1.10 ± 0.13	1.05 ± 0.10
Brain	1.94 ± 0.07	1.95 ± 0.05	1.95 ± 0.07	1.94 ± 0.06
Liver	8.44 ± 0.98	8.49 ± 1.14	8.48 ± 1.01	8.64 ± 0.90
	Relative weight, g/100 g body weight			
Ovary	0.037 ± 0.004	0.035 ± 0.006	0.041 ± 0.006	0.038 ± 0.008
Adrenal	0.028 ± 0.005	0.028 ± 0.003	0.030 ± 0.003	0.029 ± 0.004
Spleen	0.244 ± 0.025	0.220 ± 0.033	0.222 ± 0.029	0.212 ± 0.028
Kidney	0.846 ± 0.035	0.839 ± 0.043	0.885 ± 0.058	0.866 ± 0.039
Heart	0.419 ± 0.030	0.409 ± 0.034	0.446 ± 0.053	0.433 ± 0.043
Brain	0.786 ± 0.052	0.795 ± 0.057	0.789 ± 0.050	0.801 ± 0.058
Liver	3.40 ± 0.25	3.42 ± 0.21	3.41 ± 0.23	3.56 ± 0.23

*Data were expressed as mean ± SD (n = 10)*.

### GKSS as a Novel Food Material for Spontaneous Sleep

**Figure 2:**
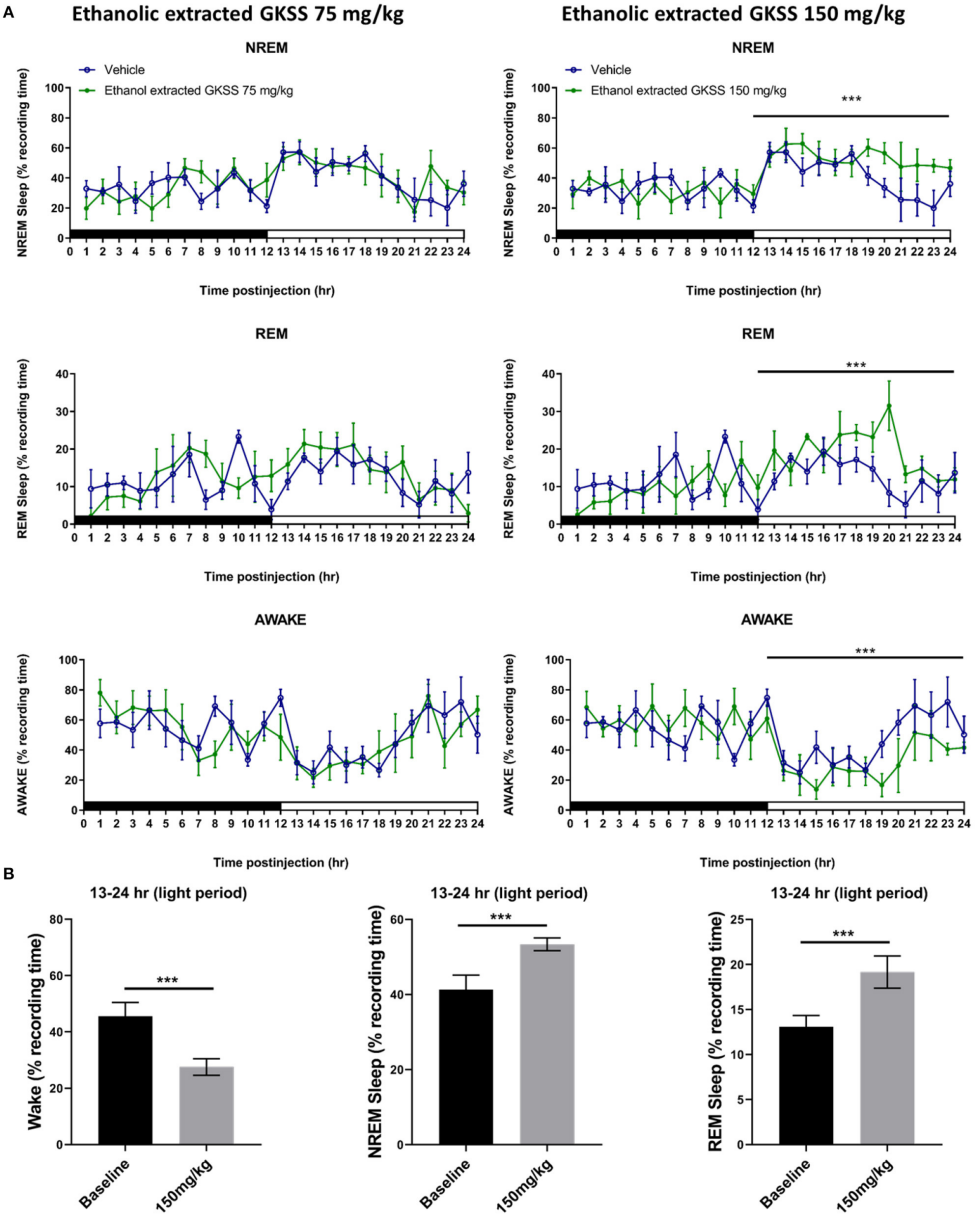
**(A)** Time-course changes and **(B)** total amounts of REM, NREM sleep, and awake produced in male Sprague-Dawley rats after 28 days daily oral administration of GKSS at 75 mg/kg and 150 mg/kg. The black bar and the white bar on the x-axes indicate the 12 h dark and 12 h light periods, respectively. Values are presented as means ± SEM (*n* = 6). ***p < 0.001 indicates significant differences when compared with the vehicle group.

To evaluate the effects of GKSS ethanolic extract on spontaneous sleep-wake activity, two doses, 75 mg/kg and 150 mg/kg, were administered 20 min prior to the dark period of the 12:12h L:D cycle. Results indicated that 75 mg/kg of GKSS ethanolic extract does not affect wakefulness, NREM sleep, and REM sleep during both the dark period and the light period (Figure 2A). However, high dose GKSS ethanolic extract (150 mg/kg) significantly enhanced NREM sleep during hours 13–24 during the 12-h light period (*p* < 0.05; Figure 2A). Moreover, REM sleep was also increased after administration of 150 mg/kg of GKSS ethanolic extract during hours 13–21 of the light period (*p* < 0.05; Figure 2A). Alternatively, the awake was mirrored in response to the summation of NREM sleep and REM sleep (*p* < 0.05; Figure 2A). The percentage of time spent in awake decreased from 43.6 ± 3.6% obtained from vehicle to 27.4 ± 2.8% during hours 13–24 (*p* < 0.05; Figure 2B). Likewise, the percentage of time spent in NREM and REM sleep increased from 42.8 ± 2.8% obtained from vehicle to 53.5 ± 2.4% during hours 13–24 and from 13.7 ± 1.2% obtained after vehicle to 19.1 ± 1.3% during hours 13–24, respectively (*p* < 0.05; Figure 2B).

### GKSS Mediated Sleep Through Nrf2 Signaling Pathway

**Figure 3:**
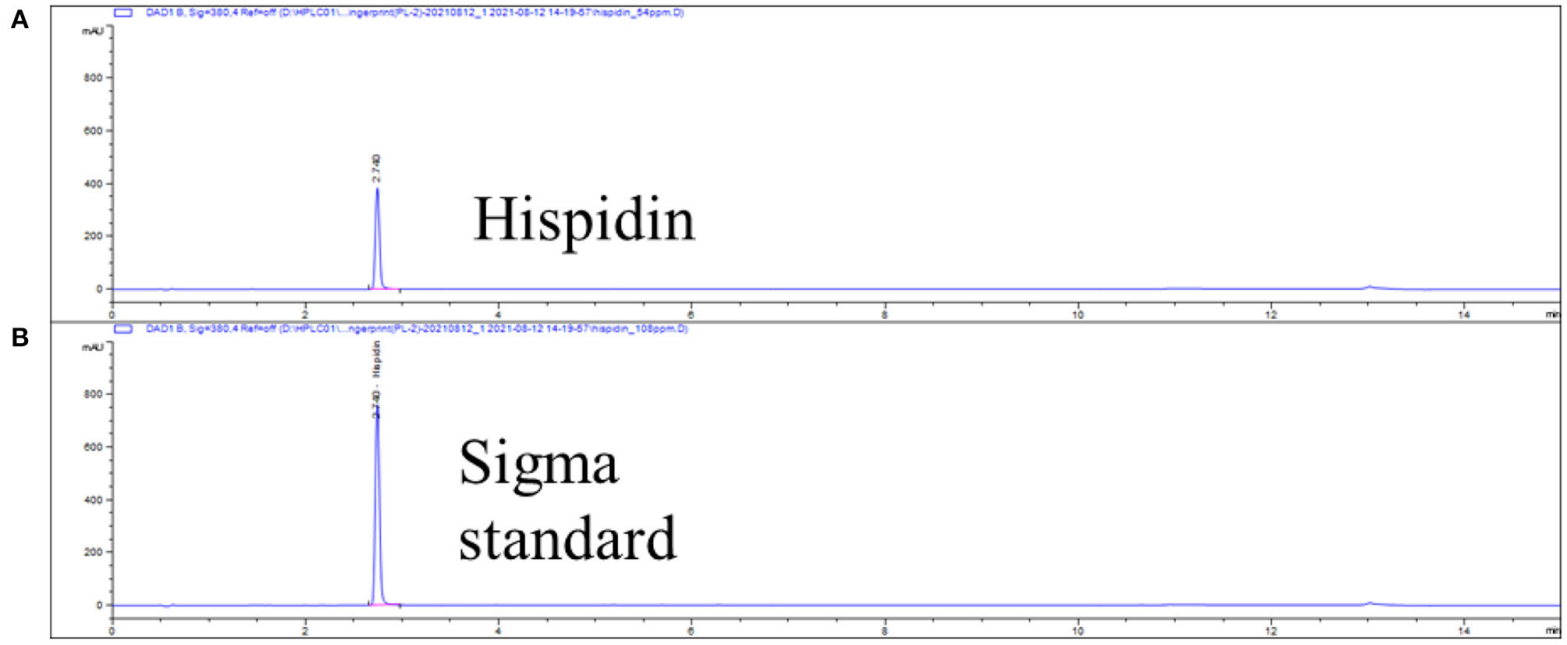
UPLC chromatographs for the identification of hispidin. The retention time of 3.36 min represents the presence of hispidin isolated from **(A)** GKSS and confirmed by the **(B)** Sigma standard (100 ppm).

**Figure 4:**
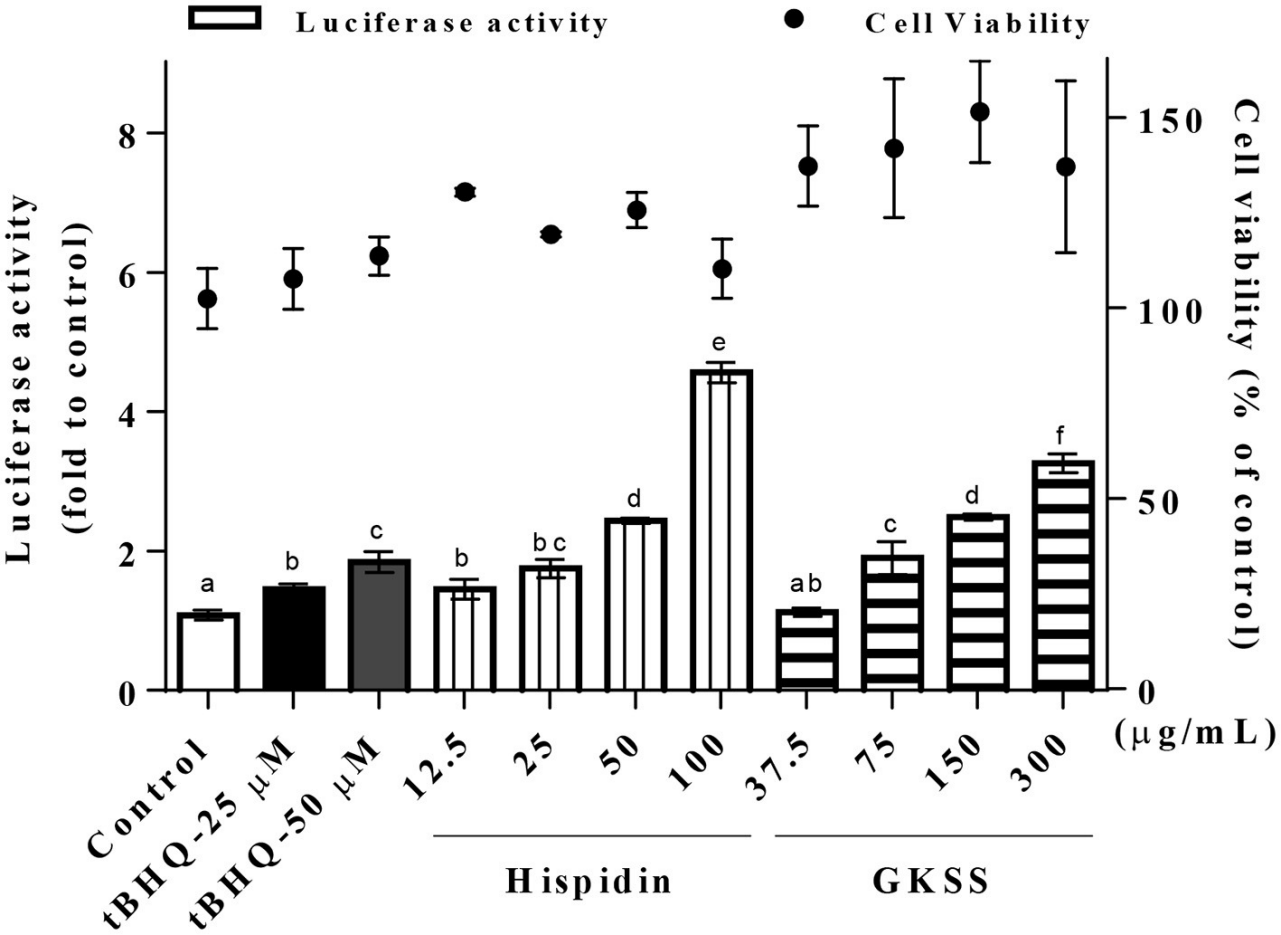
Effects of hispidin and GKSS ethanolic extract on ARE-driven luciferase reporter activity in HSC-3 cells. Luciferase activity and cell viability were assayed in parallel as described under Materials and Methods. Cells were treated with various concentrations of hispidin, GKSS ethanolic extract and t-BHQ (positive control) for 24 h. Values are mean ± SD and analyzed by one-way ANOVA with Tukey's multiple comparisons *post hoc* test. Different letters indicate statistically significant differences at *p* < 0.05.

Hispidin was first determined by comparison of UPLC retention time with that of the authentic compound purchased from Sigma (H5257, Sigma, MO, USA) as shown in Figure 3. Various concentrations of hispidin (12.5, 25, 50, and 100 μg/mL) and GKSS (37.5, 75, 150, and 300 μg/mL) were applied to the ARE-luciferase reporter system to investigate their Nrf2/ARE activation abilities (Figure 4). Parallel cell viability assays revealed no obvious cytotoxic effects from these treatments. After the cells were treated with test samples at the indicated concentration for 24 h, both hispidin and GKSS significantly induced ARE-luciferase activity in a dose-dependent manner (*p* < 0.05). Hispidin and GKSS treatment can reach as high as 4.23 and 3.02-fold increases in ARE-driven luciferase activity, respectively, suggesting that they are potential Nrf2 activators, with effects even greater than that of positive control t-BHQ (1.35 ± 0.07 and 1.71 ± 0.14 at 25 and 50 μM, respectively).

### GKSS Mediated Sleep Through Anti-inflammatory Cytokines

**Figure 5:**
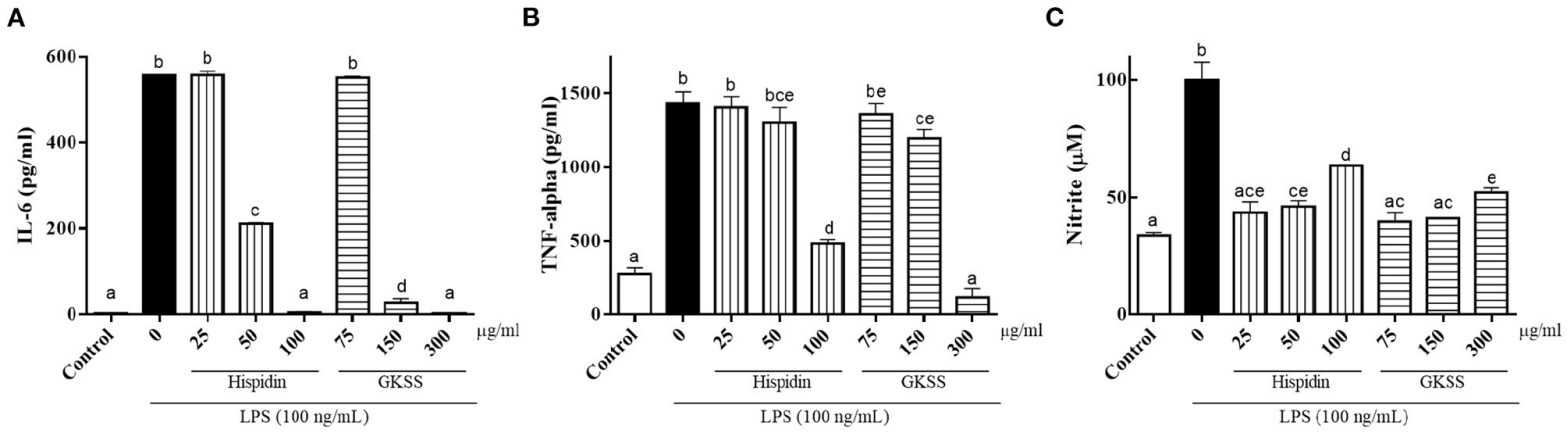
Effects of hispidin and GKSS on LPS-induced **(A)** IL-6, **(B)** TNF-α, and **(C)** NO production. RAW 264.7 cells were pretreated with hispidin and GKSS for 1 h before treatment with 100 ng/mL LPS. After incubation for 24 h, IL-6, TNF-α and NO production was detected by ELISA. The values are presented as means ± SD and analyzed by one-way ANOVA with Tukey's multiple comparisons *post hoc* test. Different letters indicate statistically significant differences at *p* < 0.05.

For hispidin concentrations ranging from 25–100 μg/mL and GKSS concentrations ranging from 75–300 μg/mL, RAW267.4 cell viability was not significantly changed after 24 h treatment with 100 ng/mL LPS compared to control group cells (data not shown). To confirm the effect of hispidin and GKSS on IL-6 (Figure 5A) and TNF-α (Figure 5B) production in LPS-induced RAW 267.4 cells, enzyme-linked immunosorbent assays (ELISA) were applied. LPS treatment significantly increased (*p* < 0.05) the IL-6 and TNF-α levels by 202.4- and 5.2-fold, respectively. However, the LPS-induced increases were dose-dependently reversed by hispidin and GKSS treatments. Results showed that 50–100 μg/mL hispidin and 150–300 μg/mL GKSS significantly suppressed the production of IL-6 while 100 μg/mL hispidin and 300 μg/mL GKSS significantly suppressed the release of TNF-α when compared to LPS-stimulated control cells. Next, the effects of hispidin and GKSS on NO production in the supernatant of LPS-treated RAW 264.7 cells were measured via Griess reagent assay. Treatment with hispidin and GKSS increased NO production in a dose-dependent manner, suggesting their color may potentially interfere with the detecting assays (Supplementary Figure 1). Despite color interference, both hispidin at 25–100 μg/mL and GKSS at 75–300 μg/mL significantly inhibited NO production in RAW 264.7 cells stimulated with LPS (*p* < 0.05; Figure 5C).

## Discussion

Growing evidence has suggested that after the COVID-19 crisis, a wide range of psychological outcomes including fear, anxiety, depression, or insecurity have been observed (30). In humans, these symptoms have been associated with changes in the circadian rhythms, which in turn has been associated with increased waking during sleep and less deep sleep (31). As a result of the effects on sleep restriction, many serious diseases and health conditions have been reported. Increased autonomic sympathetic activation, somatic problems, reduced quality of life, emotional distress, mood disorders, and behavior problems have been observed as adverse short-term health consequences, while hypertension, dyslipidemia, cardiovascular disease, weight-related issues, metabolic syndrome, type 2 diabetes mellitus, cancers, and even death have been found as adverse long-term health consequences (32). Considering that sleep deprivation can cause detrimental impacts on physical health, this study aims to test the efficacy of GKSS as a novel intervention to improve sleep health.

In this study, treatment with GKSS at 150 mg/kg significantly decreased the wakefulness, and increased total sleeping time, REM and NREM sleep in electroencephalogram (EEG) of rats. To elucidate the potential mechanisms of actions, sleep enhancement and its correlation with oxidative stress is first investigated. Nuclear factor erythroid 2-related factor 2 (Nrf2) has been known as a vital signaling pathway that regulates antioxidant genes and serves as an important bridge between the molecular clock and metabolism (33). According to a recent study, sleep deprivation for 5 consecutive days markedly reduced the protein expression level of Nrf2 in the hippocampus of rats (34). Moreover, loss of Nrf2 function in mouse fibroblasts, hepatocytes and liver can alter circadian gene expression and rhythmicity, which can disrupt normal sleep patterns and impact the timing and length of sleep (35). According to our previous study, *Sanghuangporus sanghuang* mycelium can regulate Nrf2-associated pathways and protect liver cells from paracetamol-induced hepatotoxicity by inhibiting oxidative stress (36). Moreover, the yellow polyphenol pigment hispidin found in *Sanghuangporus sanghuang* mycelium, was found to enhance the expression of Nrf2 in a dose-dependent manner (37). Consistent with these studies, this study showed that both hispidin and GKSS can induce ARE-driven luciferase activity in a concentration-dependent manner. In addition, after quantification using HPLC, the amount of hispidin in GKSS is about 25 % (data not shown), indicating that hispidin may be the key molecule in inducing Nrf2/ARE activation and the antioxidant defense machinery. Taken together, GKSS enriched with 3 mg/g hispidin in this study may link metabolism signals to the ticking of the circadian clock to potentially regulate sleep via Nrf2 signaling pathway.

Nrf2 not only plays a pivotal role in controlling the expression of antioxidant genes but also regulates anti-inflammatory gene expression and inhibits the progression of inflammation (38). One of the mechanisms underlying Nrf2-mediated anti-inflammation is through upregulation of numerous antioxidant genes. Another mechanism is that Nrf2 binds to the proximity of the proinflammatory cytokine genes, including IL-6 and IL-1b, and inhibits LPS-induced expression of these genes (39). In this study, the LPS-induced IL-6 and TNF-α increases in RAW 264.7 cells were dose-dependently reversed by hispidin and GKSS treatments. Moreover, both hispidin at 25–100 μg/mL and GKSS at 75–300 μg/mL significantly inhibited NO production in RAW 264.7 cells stimulated with LPS. Results of the present study were similar to our prior study (40), which showed that LPS-induced acute lung injury mice administered *Sanghuangporus sanghuang* mycelium could significantly decrease concentrations of TNF-α, IL-1β, IL-6, and NO in bronchoalveolar lavage. As numerous studies support that sleep loss is a factor to induce cellular and molecular inflammation (41), hispidin and GKSS possessing anti-inflammatory activities may play a pivotal role in coordinating sleep deprivation-induced inflammation.

Besides changes in sleep is driven by inflammatory mediators such as cytokines, sleep is also closely related to neurotransmitters such as norepinephrine, dopamine, histamine, and serotonin. For example, dopamine is a major regulator of sleep/wake states, and enhancement of dopaminergic neurotransmission could dramatically reduce sleep and arousal threshold and increase locomotor activity (42). In addition, noradrenergic and histaminergic activity could promote an aroused waking state, while GABAergic and serotonergic neurotransmission could promote sleep (43). Although the current study demonstrated hispidin and GKSS in regulating sleep via antioxidant and inflammatory activities, it remains to be determined whether this role extends to regulating neurotransmitter release in brain areas. In addition, how GKSS and hispidin exhibit physiological functions remain ambiguous and not fully elucidated. It is also unclear of their potential metabolism by the gut microbiota. As these are major limitations in this study, future research will be conducted to clarify the role of GKSS and hispidin in these areas.

In addition to efficacy data, providing adequate safety data is of paramount importance for both the regulatory bodies and the consumers. As the name *Phellinus linteus* has been incorrectly designated and *Sanghuangporus sanghuang* became the scientific binomial for the “sanghuang” species, there is a need to conduct safety studies for the true sanghuang strains for human consumption. In this study, safety monitoring of GKSS supplementation is assured. No unexpected patterns of concern or mortality were found for all GKSS groups. Despite a significant change in feed consumption was observed in the high-dose male group at week 3, this difference does not correlate with body weight changes and hence was not attributed to GKSS. Moreover, treatment of GKSS showed that the most obvious effect in the hematological results was a significant reduction of the platelet count in males, but such changes were not observed not in females. Even decreased platelet suggest that the GKSS may have an inhibitory effect on thrombopoietin (44), such alterations were noted to be within the normal range (529-1383 × 10^3^/μL) (45) and changes in relative spleen weight did not show a dose-dependent decrease, suggesting that these variations were less likely to be an effect of treatment.

Serum chemistry during treatment showed relatively lowered serum sodium and chloride values in the low-dose male group and relatively lowered cholesterol levels in the mid-dose female group when compared to that of the vehicle control group. However, due to the lack of dose-response, inconsistency across sexes, and absence of associated clinical or pathological findings, these differences could be considered as normal biological variations. These cumulative toxicological results indicated that 28-day oral administration of GKSS at an oral dose of up to 5 g/kg produced no adverse effects in rats. Therefore, the no observed adverse effect level of GKSS is higher than 5 g/kg in rats. However, more research such as chronic toxicity and teratogenicity is warranted to predict the risk of adverse effects resulting from the exposure of humans.

## Data Availability Statement

The original contributions presented in the study are included in the article/Supplementary Material, further inquiries can be directed to the corresponding author/s.

## Ethics Statement

The animal study was reviewed and approved by the Institutional Animal Care and Use Committee (IACUC) No. 102-9n for 28-day oral toxicity study and NTU107-EL-00182 for sedative activity.

## Author Contributions

All authors listed have made a substantial, direct, and intellectual contribution to the work and approved it for publication.

## Conflict of Interest

Grape King Bio Inc. provided support in the form of salaries for the I-CL, H-TC, T-JL, and C-CC and research materials, but did not have any additional role in the study design, data collection and analysis, decision to publish, or preparation of the manuscript. The remaining authors declare that the research was conducted in the absence of any commercial or financial relationships that could be construed as a potential conflict of interest.

## Publisher's Note

All claims expressed in this article are solely those of the authors and do not necessarily represent those of their affiliated organizations, or those of the publisher, the editors and the reviewers. Any product that may be evaluated in this article, or claim that may be made by its manufacturer, is not guaranteed or endorsed by the publisher.
