# Supplementary material for: Pilot Study: Nutritional and Preclinical Safety Investigation of Fermented Hispidin-Enriched Sanghuangporus sanghuang Mycelia: A Promising Functional Food Material to Improve Sleep
Source: Front Nutr. 2022 Jan 17;8:788965. doi: 10.3389/fnut.2021.788965 (PMC8801445; doi:10.3389/fnut.2021.788965)
Supplement: Supplementary file 1 [file Data_Sheet_1.pdf]

Figure S1

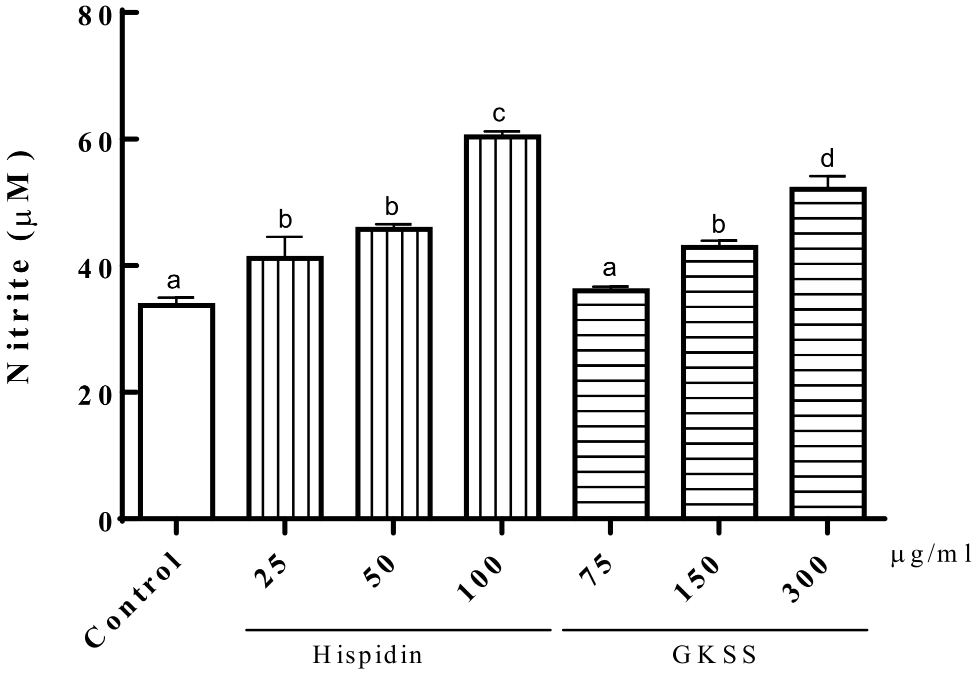

Figure S1. Dose-dependent hispidin and GKSS in NO production. Values are mean ± SD and analyzed by one-way ANOVA with Tukey's multiple comparisons post hoc test. Different letters indicate statistically significant differences at $p < 0.05$.

Table S1 Mortality and Clinical Signs of male and female rats after 28 days daily oral administration of GKSS

	Mortality (death/total)		Clinical signs	
**Group**	**Male**	**Female**	**Male**	**Female**
**Control (distilled water)**	0/10	0/10	None	None
**GKSS 1 g/kg**	0/10	0/10	None	None
**GKSS 2 g/kg**	0/10	0/10	None	None
**GKSS 5 g/kg**	0/10	0/10	None	None
